# Editorial: Rising stars in molecular and cellular oncology 2022

**DOI:** 10.3389/fonc.2023.1136335

**Published:** 2023-01-23

**Authors:** Gunjan Sharma, Jessica Dal Col, Jawed Akhtar Siddiqui

**Affiliations:** ^1^ Department of Biochemistry and Molecular Biology, University of Nebraska Medical Center, Omaha, NE, United States; ^2^ Department of Medicine, Surgery and Dentistry “Scuola Medica Salernitana, University of Salerno, Salerno, Italy; ^3^ Fred and Pamela Buffett Cancer Center, University of Nebraska Medical Center, Omaha, NE, United States

**Keywords:** cancer, pancreatic cancer, rising star, breast cancer, hepatocellular cancer (HCC)

It is our pleasure to write the editorial on the Research Topic entitled Rising Stars in Molecular and Cellular Oncology 2022 of the Frontiers in Oncology. This editorial abridges unique mixes of four original research and two review articles. The circulating tumor cells have emerged as noninvasive prognostic and diagnostic markers for many cancers (1–3).

In this issue, one review published by Huang et al. summarizes diverse functions of Four and a half LIM domains 3 (FHL3) protein in numerous cancers, including hepatocellular carcinoma (HCC), breast cancer, gastric cancer, pancreatic ductal adenocarcinoma, and non‐small cell lung cancer. FHL3 can act as a tumor suppressor or oncoprotein by up and down-regulation in many cancers. Briefly, in HCC and breast carcinoma, downregulated FHL3 impacts cell cycle proteins and acts as a tumor suppressor gene by inhibiting HIF1α, cyclin D1 and B1, and SOX4. However, upregulated FHL3 functions as an oncogene by targeting epithelial-mesenchymal transition (EMT), hypoxia, and metabolic proteins for tumor promotion, invasion, and metastasis of several types of cancer where AKT, GSkβ, and TGFβ are actively involved. The remaining five publications on this research topic are divided and discussed according to the cancer types.

## Breast cancer

Alzahayqa et al. enlightened the multifaceted role of a hydroxylase enzyme TET1 as a tumor suppressor but also as an oncogene in breast carcinoma. They have shown the distinct expression pattern of short and long isoforms of TET1 with cytoplasmic and nuclear localization, respectively. Some hormones, such as Estrogen and Gonadotrophin Releasing Hormone (GnRH), downregulate the expression of TET1 long isoform, while overexpression suppresses the oncogenic phenotypes. The expression of the short TET1 is elevated in the luminal breast cancer model, whereas both isoforms are depleted in basal breast cancer.

Muraro et al. anticipated the clinical importance of analysis of circulating tumor cells (CTCs) coupled with tumor- and antigen-specific T-cell immunity through a liquid biopsy approach. They found and correlated the high level of clonality of TCR repertoire in the peripheral blood of the patients responding to therapy, suggesting that the CTCs and anti-tumor T-cell immunity could be exploited as an immune-oncological biomarker. In addition to prognosis, the immunotherapeutic outcome of metastatic breast cancer patients can be improved by using this promising tool as predictive biomarker.

## Pancreatic adenocarcinoma

In continuation of identifying a prognostic biomarker through a non-invasive approach, Guan et al. found circulating tumor DNA (ctDNA) as a valuable prognostic biomarker in metastatic pancreatic adenocarcinoma (mPAC). 425-gene capture panel next-generation sequencing (NGS) of ctDNA collected from 40 tumor tissue and 35 blood samples of mPAC patients revealed a significant correlation between ECOG score, CA19-9, KRAS mutation, and overall survival. Besides, CA19-9, CDKN2A, or SMAD4 mutation in ctDNA are highly associated with progression-free survival of the mPAC patients. Conclusively, ctDNA can be used as an accurate predictive tool in mPAC patients.

## Hepatocellular carcinoma

Xiao et al. identify three cuprotosis-related genes (CRGs) as strong and early detection markers of HCC. They constructed a predictive prognostic model based on clinical information of HCC patients from the GEO and TCGA databases and validated by internal and external validation sets. During the analysis of HCC patients’ data sets, they observed that cuprotosis-mediated patterns-related genes (CMPRGs) control several regulatory mechanisms that influence the prognosis, clinicopathological conditions, and the amount of tumor-infiltrating immune cells in HCC patients. CMPRG_score seems to be a specific and sensitive prognostic marker and can be helpful in the management of targeted immunotherapy for HCC patients.

Moreover, the review of De Re et al. elaborated on various types of intrinsic cell death and different immune responses with prognoses in HCC. They summarized the significant differences between apoptosis, autophagy, and necrosis. In addition, during several types of cell death, heat shock proteins (HSPs), ficolin 3, and several other molecules such as ATP, DNA, and RNA can function as damage-associated molecular patterns (DAMPs) and promote an anti-tumor immune response in HCC patients.

Altogether, the regulation and the functions of TET1 isoforms and FHL3 in different human cancers may help to develop novel targeted therapeutics. On the other hand, the clinical relevance of the evaluation of CTCs and ctDNA and their use as a prognostic biomarker in metastatic conditions could improve the management of cancer patients. Also, the identification and the pharmacological induction of specific cell death pathways-related genes can activate/regulate the immune cells in the tumor microenvironment improving tumor immunosurveillance.

## Author contributions

GS and JAS wrote the initial draft. All authors listed have revised and approved the final version for publication.
